# Interactions between IL-32 and tumor necrosis factor alpha contribute to the exacerbation of immune-inflammatory diseases

**DOI:** 10.1186/ar2074

**Published:** 2006-11-01

**Authors:** Hirofumi Shoda, Keishi Fujio, Yumi Yamaguchi, Akiko Okamoto, Tetsuji Sawada, Yuta Kochi, Kazuhiko Yamamoto

**Affiliations:** 1 Department of Allergy and Rheumatology, Graduate School of Medicine, University of Tokyo, 7-3-1 Hongo, Bunkyo-ku, Tokyo 113-0033, Japan; 2 Laboratory for Rheumatic Diseases, SNP Research Center, RIKEN, 1-7-22 Suehiro-cho, Tsurumi-ku, Yokohama, Kanagawa 230-0045, Japan

## Abstract

IL-32 is a newly described cytokine in the human found to be an *in vitro *inducer of tumor necrosis factor alpha (TNFα). We examined the *in vivo *relationship between IL-32 and TNFα, and the pathologic role of IL-32 in the TNFα-related diseases – arthritis and colitis. We demonstrated by quantitative PCR assay that IL-32 mRNA was expressed in the lymphoid tissues, and in stimulated peripheral T cells, monocytes, and B cells. Activated T cells were important for IL-32 mRNA expression in monocytes and B cells. Interestingly, TNFα reciprocally induced IL-32 mRNA expression in T cells, monocyte-derived dendritic cells, and synovial fibroblasts. Moreover, IL-32 mRNA expression was prominent in the synovial tissues of rheumatoid arthritis patients, especially in synovial-infiltrated lymphocytes by *in situ *hybridization. To examine the *in vivo *relationship of IL-32 and TNFα, we prepared an overexpression model mouse of human IL-32β (BM-hIL-32) by bone marrow transplantation. Splenocytes of BM-hIL-32 mice showed increased expression and secretion of TNFα, IL-1β, and IL-6 especially in response to lipopolysaccharide stimulation. Moreover, serum TNFα concentration showed a clear increase in BM-hIL-32 mice. Cell-sorting analysis of splenocytes showed that the expression of TNFα was increased in resting F4/80^+ ^macrophages, and the expression of TNFα, IL-1β and IL-6 was increased in lipopolysaccharide-stimulated F4/80^+ ^macrophages and CD11c^+ ^dendritic cells. In fact, BM-hIL-32 mice showed exacerbation of collagen-antibody-induced arthritis and trinitrobenzen sulfonic acid-induced colitis. In addition, the transfer of hIL-32β-producing CD4^+ ^T cells significantly exacerbated collagen-induced arthritis, and a TNFα blockade cancelled the exacerbating effects of hIL-32β. We therefore conclude that IL-32 is closely associated with TNFα, and contributes to the exacerbation of TNFα-related inflammatory arthritis and colitis.

## Introduction

Tumor necrosis factor alpha (TNFα) is a potent proinflammatory cytokine and is related to several inflammatory diseases such as rheumatoid arthritis (RA) and inflammatory bowel diseases (IBDs). RA is a persistent inflammatory arthritis and is thought to be an autoimmune disease. Inflammation of the joints results in the destruction of cartilage and bone early in the course of the disease. Although the pathogenesis of RA is still unclear and may be heterogeneous, several proinflammatory cytokines participate in promoting the inflammation of the joints. TNFα facilitates arthritis and the destruction of bone [1-4]. TNFα is secreted by several kinds of inflammatory cells, including macrophages, monocytes, T cells, and synovial fibroblasts. TNFα induces other inflammatory cytokines and promotes osteoclastogenesis to destroy the bones. TNFα transgenic mice develop inflammatory arthritis spontaneously [1]. Moreover, TNFα inhibition decreases the severity of arthritis, and both monoclonal antibodies to TNFα and a soluble tumor necrosis factor receptor analog have been used as effective therapies for RA and for other types of inflammatory arthritis [5-8]. In addition, other cytokines, such as IL-1 and IL-6, are also known to be important participants, and the inhibition of these cytokines has been a part of the effective therapies for RA in clinical practice [4].

TNFα plays a pivotal role in the pathogenesis of IBDs including Crohn's disease. The murine model of IBD, trinitrobenzen sulfonic acid (TNBS)-induced colitis, is exacerbated in TNFα transgenic mice [9], and is ameliorated in tumor necrosis factor receptor 2-knockout mice [10]. In the clinical setting, TNFα blockade by infliximab is demonstrated as a useful therapy for Crohn's disease [11]. The mechanisms of TNFα production in these inflammatory diseases, however, remain to be clarified.

Human IL-32 (hIL-32) has been reported as a novel cytokine. IL-32 was cloned as a gene induced by IL-18 and was formerly known as natural killer cell transcript 4 [12,13]. IL-32 induces TNFα secretion in human monocyte and mouse macrophage cell lines. hIL-32 has four splice variants, IL-32α, IL-32β, IL-32γ, and IL-32δ. IL-32α is present in intracellular locations, and IL-32β is secreted from the cells. IL-32α and IL-32β are thought to be the major expressed variants. The sequences of IL-32β and IL-32γ are quite similar. A mouse homolog of IL-32 has not so far been reported.

IL-32 is expressed in lymphoid tissues, such as the thymus, the spleen, and the intestines. Human natural killer cells increase the secretion of IL-32 by IL-18 + IL-12 stimulation, and human peripheral blood mononuclear cells (PBMCs) also secrete IL-32 after stimulation with concanavalin A (Con A). The fact that the IL-32-related cytokines, TNFα and IL-18, show a close correlation with arthritis [14,15] implies that IL-32 has a pathologic role in inflammatory diseases. Indeed, the expression of IL-32 is increased in synovial tissues from RA patients, and the administration of recombinant IL-32γ into mice joints provokes cellular infiltration in the joint spaces [16]. We choose IL-32β for our assay, because IL-32β was reported as a dominant variant and as a secreted protein from the cells, and the sequences of IL-32β and IL-32γ were basically similar [13].

We demonstrated that IL-32 is expressed in various lymphoid cells, and in the synovial-infiltrated lymphocytes of RA patients. *In vivo*, we prepared overexpression model mice of human IL-32β by bone marrow transplantation (BM-hIL32). The expression and secretion of TNFα were increased in resting F4/80^+ ^splenic macrophages of BM-hIL-32 mice, and the expression and secretion of TNFα, IL-1β, and IL-6 were increased in F4/80^+ ^splenic macrophages and CD11c^+ ^splenic dendritic cells after lipopolysaccharide (LPS) stimulation. In fact, the murine models of TNFα-related diseases, TNBS-induced colitis and collagen antibody-induced arthritis, were exacerbated in BM-hIL-32 mice. Furthermore, hIL-32β-transduced CD4^+ ^T cells showed marked exacerbation of collagen-induced arthritis, an effect that was, in part, cancelled by TNFα blockade. Our data indicate that IL-32 is closely associated with TNFα and that it plays a role in the exacerbation of inflammatory diseases.

## Materials and methods

### Mice

DBA/1J mice and C57BL/6 mice were obtained from Japan SLC (Shizuoka, Japan). All mice were used at 6–8 weeks of age. All animal experiments were conducted in accordance with institutional and national guidelines.

### Collagen-induced arthritis and collagen antibody induced arthritis

Collagen-induced arthritis was induced as described previously [17]. In short, bovine type II collagen (Chondrex, Redmond, WA, USA) was emulsified with an equal volume of Complete Freund's adjuvant (Chondrex). DBA/1J mice were immunized with 50 μg bovine type II collagen intradermally at the base of the tail on day 0 and day 21. Collagen antibody-induced arthritis was induced by intravenous injection of 2 mg arthrogen mAb cocktail to type II collagen, and 3 days later by intraperitoneal injection of 50 μg LPS (Chondrex), as described previously [18]. The arthritis score was determined by erythema, swelling, or ankylosis per paw, as described elsewhere [19]. In some experiments, 50 μg/day etanercept (Wyeth, Madison, NJ, USA) was administered intraperitoneally for 14 days after CD4^+ ^T-cell transfer. The antiarthritic effect of human tumor necrosis factor receptor Fc fusion protein (etanercept) was demonstrated in collagen-immunized mice [8]. Sacrifice was performed 40 days after the first immunization in collagen-induced arthritis mice.

### Trinitrobenzen sulfonic acid-induced colitis

TNBS (Wako, Osaka, Japan) was diluted to a final concentration of 1.75% with 50% ethanol and PBS. C57BL/6 mice were anesthetized with 500 μg nembutal (Dainippon Pharmaceutical, Osaka, Japan) by intraperitoneal injection, and 100 μl (1.75 mg) TNBS was administered into the rectum through a 4 cm inserted catheter, as previously described [10]. The body weight was measured daily, and mice were sacrificed 4 days after induction for further analysis. One group of BM-hIL-32 mice were administered 200 μg/day etanercept (Wyeth) intraperitoneally after induction of colitis; other mice were administered the same volume of PBS each day.

### Cytokines and cell lines

Recombinant human TNFα, IL-12, IL-18, IL-23, granulocyte-macrophage colony-stimulating factor, and IL-4 were obtained from R&D Systems (Minneapolis, MN, USA). The human 293T cell line and the mouse macrophage cell line, Raw 267.4, were obtained from ATCC (Manassas, VA, USA). Cell lines and primary cells were cultured with RPMI 1640 medium supplemented with 10% FCS, 2 mM γ-glutamine, 100 U/ml penicillin, 100 μg/ml streptomycin, and 5 × 10^-5^M 2-mercapto ethanol. Recombinant human cytokines were added to the culture medium as follows: 50 ng/ml human TNFα, 50 ng/ml hIL-23, 50 ng/ml IL-18, and 10 ng/ml IL-12 (R&D Systems).

### Monoclonal antibodies and flow cytometry

Monoclonal antibodies to mouse CD3, CD4, CD8, CD11c, CD19, and F4/80 were obtained from BD Biosciences (San Jose, CA, USA). Cell sorting was performed on a FACSVantage system (Becton Dickinson Immunocytometry Systems, Mountain View, CA, USA), and analysis was performed on an EPICS flow cytometer (Beckman Coulter, Fullerton, CA, USA).

### Synovial tissue samples from rheumatoid arthritis patients

Synovial membranes and synovial fibroblasts were obtained from patients with RA satisfying the diagnostic criteria of the American College of Rheumatology [20]. We sampled pathological joint synovial tissues from individuals with RA who underwent arthroplasty surgery. Informed consent was obtained from all patients. Synovial fibroblasts were isolated as formally described [21]. In brief, the collected synovial tissues were digested with collagenase type IV, hyaluronidase, and DNase I (Sigma-Aldrich Corporate, St. Louis, MO, USA), and were passed through a metal screen to prepare isolated cells.

### Peripheral blood mononuclear cells

Human PBMCs were isolated from the leukocytes of a healthy donor by Ficoll-Paque (Amersham Pharmacia, Dübendorf, Switzerland). In some experiments, PBMCs were subjected to negative selection with MACS (magnetic-activated cell sorting) using anti-human CD3 mAb (Miltenyi Biotec, Auburn, CA, USA). PBMCs were stimulated with Con A or plate-coated anti-human CD3 antibodies and anti-human CD28 antibodies (R&D Systems). The stimulated cells were incubated for 24 hours and were separated by MACS with anti-human CD4 mAb, anti-human CD8 mAb, anti-human CD14 mAb, and anti-human CD20 mAb (BD PharMingen, San Diego, CA, USA).

Human monocyte-derived dendritic cells (MoDCs) were isolated and cultured as previously described [22]. Briefly, CD14^+ ^cells were isolated from human PBMCs by the MACS procedure and were cultured with 50 ng/ml recombinant human granulocyte-macrophage colony-stimulating factor and IL-4. After 7 days of incubation, MoDCs were cultured with 25 ng/ml LPS (Sigma) or 50 ng/ml human TNFα for 24 hours.

### Preparation of retroviral constructs of IL-32β

hIL-32β cDNA was isolated from the human cDNA library according to the reported nucleotide sequence (GenBank: NM 001012631) [13]. The full-length fragments were subcloned into the retrovirus vector pMIG [23]. In some experiments, a cell line was cultured with 1 ml of the supernatant of hIL-32β or mock-transfected (pMIG-transfected) 293T cells in the presence of 5 μg/ml polymixin B (Pfizer, New York, NY, USA) for 24 hours [24].

### Production of retroviral supernatants and retroviral transduction

Total splenocytes were cultured for 48 hours in the presence of Con A (10 μg/ml) and mIL-2 (50 ng/ml) (R&D Systems). Retroviral supernatants were obtained by transfection of pMIG or pMIG-hIL-32β into PLAT-E packaging cell lines using FuGENE 6 transfection reagent (Roche Diagnostic System, Somerville, NJ, USA) [25]. For the detection of green fluorescent protein (GFP)-positive cells, we used an EPICS flow cytometer (Beckman Coulter, Fullerton, CA, USA).

### Gene transduction to mouse splenocytes and adoptive transfer

Retroviral gene transduction was performed as described [26,27]. Briefly, Falcon 24-well plates (BD Biosciences) were coated with the recombinant human fibronectin fragment CH296 (Retronectin; Takara, Otsu, Japan). The viral supernatant was preloaded into each well of the CH296-coated plate, and the plate was spun at 2400 rpm for 3 hours at room temperature. This procedure was repeated three times. The viral supernatant was washed away, and Con A-stimulated splenocytes were placed into each well (1 × 10^6 ^per well). Cells were cultured for 48 hours to allow infection to occur [23,28].

A CD4^+ ^T-cell population was prepared by negative selection by MACS with anti-CD19 mAb, anti-CD11c mAb, and anti-CD8a mAb (BD PharMingen). The gene-transduced CD4^+ ^T cells were suspended in PBS and injected intravenously (1 × 10^7^) 23 days after the first immunization of bovine type II collagen.

### Bone marrow precursor cell isolation, infection, and transfer

Bone marrow precursor cell isolation, retrovirus infection, and transfer were performed as described previously [29]. In brief, DBA/1J mice or C57BL/6 mice were treated with 5 mg/body 5-fluorouracil (Sigma) dissolved in PBS. After 5 days, bone marrow cells were harvested and cultured with 50 ng/ml mIL-3, mIL-6, and mouse stem cell factor (R&D Systems) for 48 hours. The bone marrow cells were then spin-infected with the retrovirus supernatants using 16 μg/ml polybrene for 90 minutes at 2400 rpm and 25°C. Recipient mice, which were the same strain as the donor mice, were treated by 700 rad whole-body radiation and were injected with 1 × 10^6 ^bone marrow cells intravenously. To avoid wasting of the recipient mice due to the overexpression of inflammatory cytokine, the GFP-positive cells among the bone marrow cells were adjusted to around 10% before transplantation. Recipient mice were maintained for 6–9 weeks until analysis. In some experiments, splenocytes derived from bone marrow transplantation DBA/1J mice were cultured for 48 hours with RPMI 1640 medium containing 10% FCS and 1 μg/ml LPS (Sigma) for further analysis.

### RT-PCR and quantitative PCR

RNA of the cells was extracted using the RNeasy Micro Kit and RNeasy Mini Kit (Qiagen, Valencia, CA, USA). RNA from the tissues was isolated by the acid guanidinium thiocyanate-phenol-chloroform extraction method using ISOGEN (Nippon Gene, Tokyo, Japan). RNA was reverse-transcribed to cDNA with random primers (Invitrogen, Carlsbad, CA, USA) and Superscript III according to the manufacturer's protocol (Invitrogen). Quantitative real-time PCR analysis was performed by the Assay-on-Demand TaqMan probe (Hs00992441_m1 for natural killer cell transcript 4) using the ABI PRISM 7900 system (Applied Biosystems, Branchburg, NJ, USA) in the analysis of tissue expression, and using the iCycler system (Bio-rad, Hercules, CA, USA) in the analysis of cellular expression. The TaqMan gene expression assay was performed according to the manufacturer's protocol; a 20 μl reaction mixture contained 1 μl of 20 TaqMan gene expression assay, 9 μl cDNA template, and 10 μl of 2x TaqMan universal master mix. For analyzing cellular expression, the PCR mixture consisted of 25 μl SYBR Green Master Mix (Qiagen), 15 pmol forward and reverse primers, and cDNA samples for a total volume of 50 μl. The results of real-time PCR are shown in terms of relative expression compared with β-actin. The primers used in the real-time PCR are presented in Table 1. The indicated primers and probes for IL-32 were designed for detecting all known isoforms of hIL-32.

### Immunoassays of mouse cytokines

Concentrations of mouse TNFα, IL-1β, and IL-6 in sera and culture supernatants were measured by sandwich ELISA according to the manufacturer's protocol (BD Pharmingen). An automatic microplate reader (Bio-rad 550; Bio-rad) was used to measure the optical density.

### Histopathology

Tissue samples of RA patients and sacrificed mice were embedded in paraffin wax after 10% formaldehyde fixation and decalcification. The sections were stained with H & E. Synovial tissues were graded by mononuclear cell infiltration, by pannus formation, and by cartilage erosion as described previously [30]. Inflammation of the colon was graded by the extent, cellular infiltration, ulceration, and regeneration as described elsewhere [10].

### *In situ *hybridization

*In situ *hybridization of the synovial tissue samples was performed as previously described [31]. Single-stranded sense and antisense probes were generated by *in vitro *transcription from the cDNA encoding hIL-32β, nucleotides 30–340 (311 base pairs), which was marked by digoxinogen using the DIG RNA Labeling Mix (Roche, Basel, Switzerland). The sequence of the hIL-32 probe was complementary to the unique sequence of hIL-32β, because IL-32β is the dominant secreting isoform of IL-32. This probe could detect the cDNA of hIL-32β and IL-32γ, but not of IL-32α or IL-32δ, by Southern hybridization (data not shown). Hybridization was performed with probes at a concentration of 100 ng/ml at 60°C for 16 hours. Anti-DIG AP conjugate (Roche) was used as the detection antibody, and coloring reactions were performed with BM purple AP substrate (Roche). The sections were counterstained with Kernechtrot stain solution (Mutoh, Tokyo, Japan), were dehydrated, and were mounted with Malinol (Mutoh). We also examined control probes, which yielded no specific hybridization (data not shown).

### Statistical analysis

Data are expressed as the mean ± standard deviation. All results were obtained from at least three independent experiments. Statistical significance was determined by the Mann-Whitney U test, and *P *< 0.05 was considered significant.

## Results

### Increased IL-32 expression in activated human peripheral blood mononuclear cells

**Figure 1:**
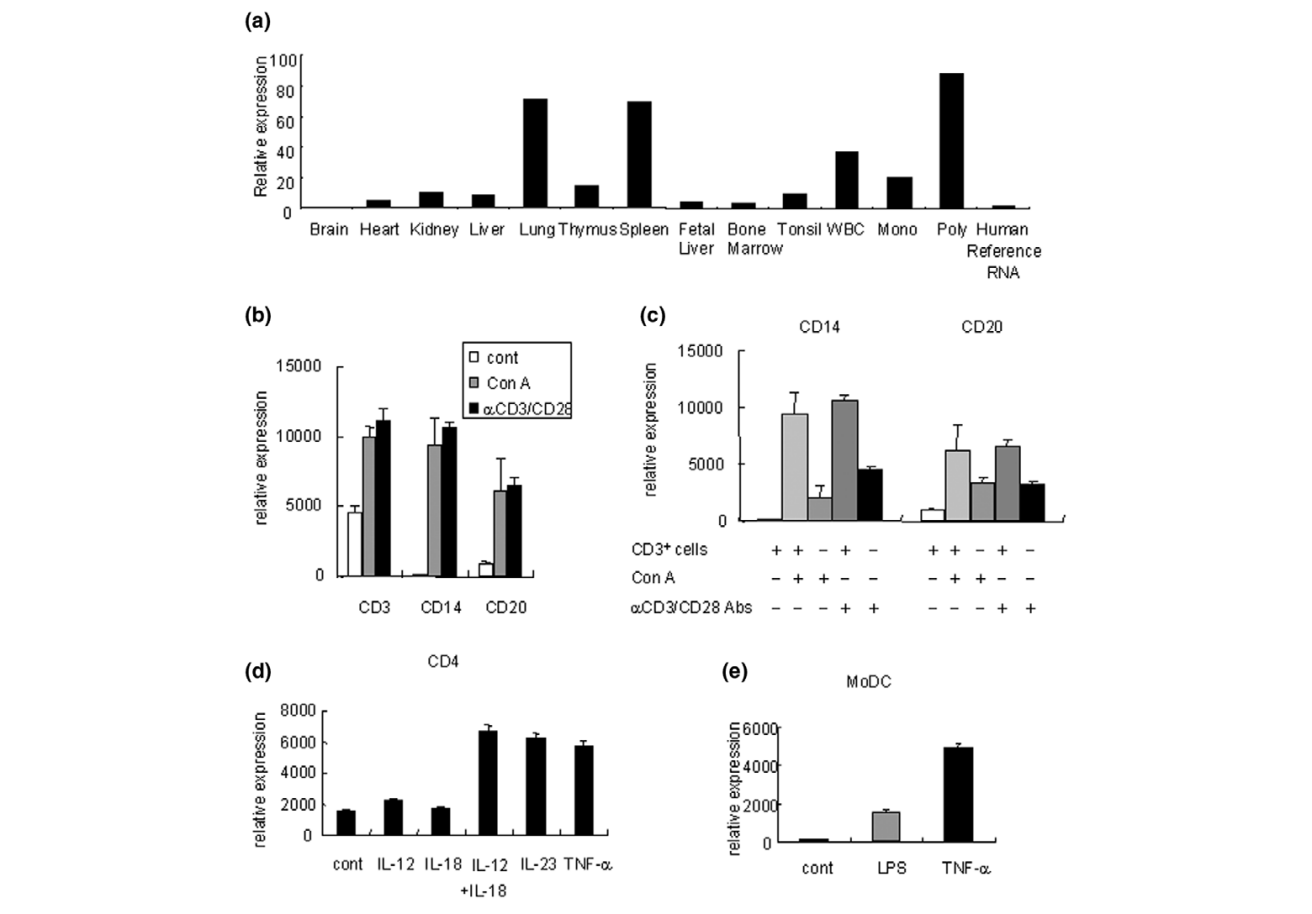
Examination of tissue and cell expression of IL-32 by quantitative real-time PCR. **(a) **Tissue expression of IL-32. WBC, white blood cells. **(b) **Human peripheral blood mononuclear cells (PBMCs) expressed IL-32. PBMCs were cultured with or without concanavalin A. PBMCs were also stimulated by immobilized anti-human CD3 and anti-human CD28 antibodies. Cont, control. **(c) **IL-32 expression of monocytes and B cells after the depletion of CD3^+ ^cells. **(d) **Peripheral CD4^+ ^T cells were cultured with the indicated inflammatory cytokines for 24 hours. **(e) **Human monocyte-derived dendritic cells (MoDCs) were cultured with lipopolysaccharide (LPS) or tumor necrosis factor alpha (TNFα) for 24 hours to induce maturation. The data are representative of at least three independent studies.

A previous study showed that IL-32 was expressed in the thymus, the spleen, the intestines, and Con A-stimulated PBMCs by northern blotting and electrochemiluminescence [13]. At first we examined the tissue and cellular expression of IL-32 by quantitative real-time PCR. The tissue expression of IL-32 was prominent in the spleen, the lung, and the peripheral white blood cells (Figure 1a). IL-32 was therefore expressed mainly in the lymphoid tissues and leukocytes.

Since human PBMCs secrete IL-32 by means of the stimulation of Con A [13], we investigated which components of PBMCs expressed IL-32 during both the resting and activated states. CD3^+ ^T cells expressed significant amounts of IL-32 without stimulation, and CD3^+ ^T cells, CD14^+ ^monocytes, and CD20^+ ^B cells increased IL-32 expression after Con A stimulation (Figure 1b). The cellular IL-32 expression was essentially the same in the case of anti-CD3 antibody and anti-CD28 antibody stimulation, which stimulated T cells specifically (Figure 1b). Monocytes or B cells, however, had lower IL-32 expression when they were cultured without CD3^+ ^T cells (Figure 1c). Activated T cells therefore have the capability of inducing IL-32 expression in monocytes and B cells.

Several dendritic cell-derived cytokines, such as IL-12, IL-18, and IL-23, are known activators of T cells and important cytokines in the pathogenesis of autoimmune diseases. CD4^+ ^T cells increased IL-32 expression in response to IL-12 + IL-18 and IL-23 stimulation (Figure 1d). In contrast, CD8^+ ^T cells did not increase IL-32 expression (data not shown). Moreover, TNFα also increased IL-32 expression significantly in CD4^+ ^T cells (Figure 1d).

We also generated human MoDCs from CD14^+ ^PBMCs. Although immature control MoDCs hardly expressed IL-32, LPS-stimulated MoDCs and, especially, TNFα-stimulated MoDCs showed a significant increase of IL-32 expression (Figure 1e). In this way, several kinds of immune cells, including T cells, B cells, monocytes, and dendritic cells, were shown to express IL-32, especially in activated states. Moreover, reciprocal IL-32 induction by TNFα was observed in CD4^+ ^T cells and MoDCs.

### Abundant IL-32 expression in the synovial-infiltrated lymphocytes of rheumatoid arthritis patients

**Figure 2:**
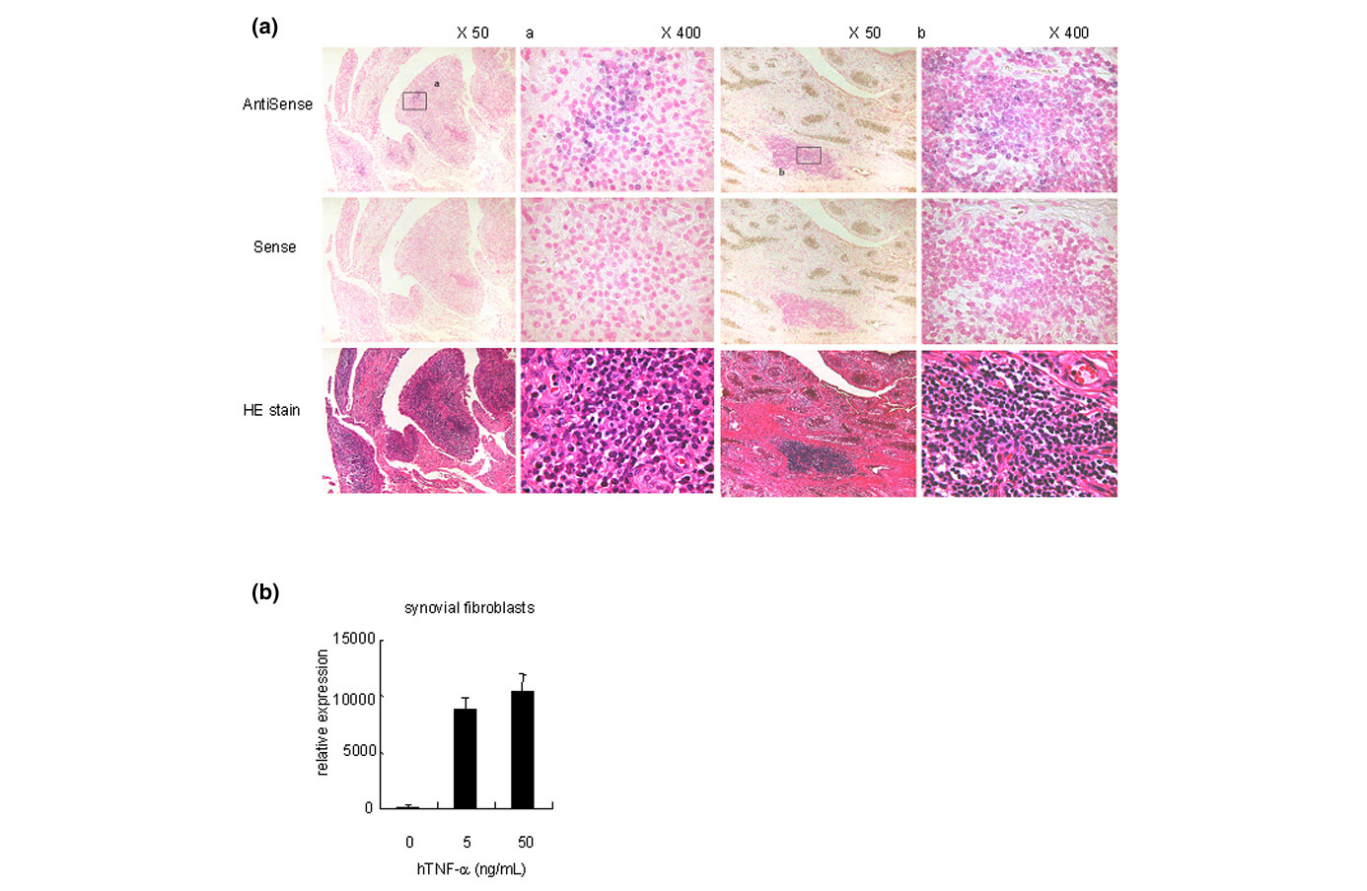
IL-32 was abundantly expressed in the synovial tissues of rheumatoid arthritis patients. **(a) ***In situ *hybridization of the synovial tissues from rheumatoid arthritis (RA) patients. IL-32β was expressed in the synovial-infiltrated lymphocytes of RA patients. HE stain, hematoxylin and eosin stain. We examined the tissue samples from four RA patients, and show representative examples. **(b) **IL-32 expression of the synovial fibroblasts derived from four RA patients in response to human tumor necrosis factor alpha (hTNFα).

To examine the pathological roles of IL-32 in RA, we tested IL-32 expression in the synovial tissues of RA patients by *in situ *hybridization (Figure 2a). We detected abundant IL-32 expression in the synovial-infiltrated lymphocytes of RA patients rather than in the synovial lining cells. We could not detect the IL-32 expression in the synovial lining layers, where monocytes and synovial fibroblasts usually exist. Synovial fibroblasts produce cytokines and proteases, which play an important role in joint inflammation [32]. We examined the IL-32 expression of the synovial fibroblasts derived from four RA patients *in vitro*. The synovial fibroblasts expressed IL-32 significantly after the stimulation of TNFα (Figure 2b). This result suggested the potential contribution of IL-32 to the joint inflammation mediated by synovial fibroblasts.

### Cytokine expression of the bone marrow chimera mice of IL-32β

**Figure 3:**
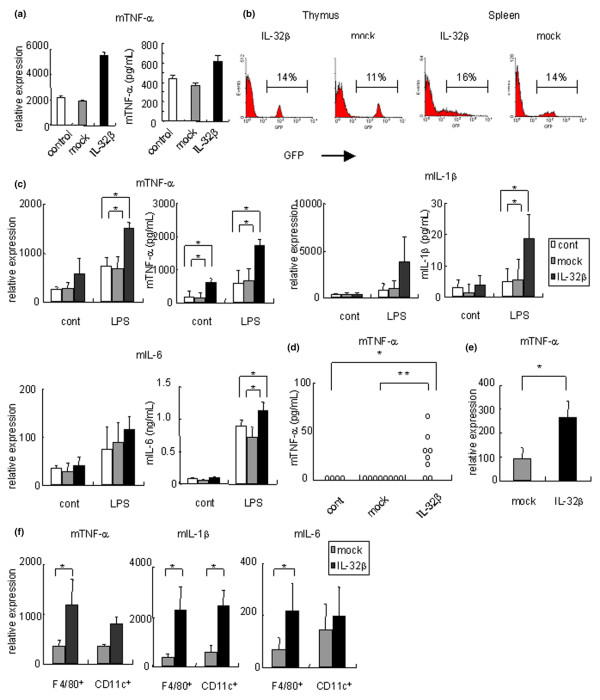
Inflammatory cytokines were induced by human IL-32β in mice. **(a) **Raw 267.4 was cultured with the supernatant of human IL-32β (h IL-32β) or mock-transfected mammalian cells (293T) for 24 hours. Left, relative expression of mouse tumor necrosis factor alpha (mTNFα), compared with β-actin; right, secreted TNFα protein level measured by ELISA. **(b) **We generated hIL-32β overexpressed mice by transplantation of hIL-32β-transduced bone marrow cells. The expression of green fluorescent protein, was analyzed by flow cytometry 6–9 weeks after transplantation. **(c) **Expression of mTNFα, mIL-1β and mIL-6 in the cultured splenocytes of the control group (white bars; *n *= 3), or bone-marrow chimeric mice of the mock group (BM-Mock mice) (gray bars; *n *= 4), or hIL-32β (BM-hIL-32) (black bars; *n *= 4) with or without 1 μg/ml lipopolysaccharide (LPS). Concentrations of indicated cytokines of the cultured supernatants are shown in the right-hand figures. **(d) **Serum concentration of mTNFα determined in control mice (*n *= 4), in BM-Mock mice (*n *= 8), and in BM-hIL-32 mice (*n *= 8). **(e) **Expression of mTNFα in splenic F4/80^+ ^CD11c^- ^macrophages of BM-Mock mice (gray bars; *n *= 4) and in BM-hIL-32 mice (black bars; *n *= 4). **(f) **Expression of mTNFα, mIL-1β, and mIL-6 in LPS-stimulated splenic F4/80^+ ^CD11c^- ^macrophages and CD11c^+^, CD3^-^, and CD19^- ^dendritic cells in BM-Mock mice (gray bars; *n *= 4), and in BM-hIL-32 mice (black bars; *n *= 4). Data are representative of at least three independent studies. *P < 0.05, **P < 0.01, BM-hIL-32 mice versus BM-Mock mice or control mice.

Activated macrophages are known to be important sources of the inflammatory cytokines in the joints of arthritis patients. hIL-32 was reported to induce TNFα in the mouse macrophage cell line Raw 267.4 [13]. We next confirmed the function of IL-32 with our retroviral construct, MIG-hIL-32β. We choose IL-32β for our assay because IL-32β was reported as a dominant variant and a secreted protein from the cells [13]. The mouse macrophage cell line Raw 267.4 was cultured with the supernatants of MIG-hIL-32β-transfected cells. After 24 hours, the mRNA expression of TNFα was increased by the stimulation of hIL-32β (Figure 3a). In addition, the protein levels of TNFα were increased in the supernatants of hIL-32β-stimulated cells (Figure 3a).

To examine the proinflammatory effect of constitutively expressed IL-32 *in vivo*, we prepared BM-hIL-32 mice. Six weeks to 9 weeks after the bone marrow transplantation, approximately 15% of the cells were GFP-positive in the thymus and the spleen of the BM-hIL-32 mice (Figure 3b). The GFP expression of CD4^+ ^cells, CD8^+ ^cells, CD11c^+ ^cells, CD19^+ ^cells, and F4/80^+ ^cells was also analyzed. There was no significant difference in specific cellular components or the percentage of GFP expression between mock mice and BM-hIL-32 mice (data not shown). hIL-32β expression in the spleen of BM-hIL-32 mice was also confirmed by quantitative real-time PCR and *in situ *hybridization (data not shown).

In accordance with the data of cell lines, freshly isolated splenocytes of BM-hIL-32 mice showed increased expression and secretion of TNFα, compared with those of BM-Mock mice (Figure 3c). We observed no increased expression and secretion of IL-1β or IL-6 in freshly isolated splenocytes of BM-hIL-32 mice. The serum concentration of TNFα protein was elevated significantly in BM-hIL-32 mice (Figure 3d). The serum concentration of IL-1β or IL-6 protein was not detected in BM-hIL-32 mice, in BM-Mock mice, or in control mice. Cell sorting analysis of splenocytes of BM-hIL-32 mice revealed that the expression of TNFα was increased in freshly isolated F4/80^+ ^macrophages (Figure 3e). Other cellular components (that is, CD4^+ ^cells, CD8^+ ^cells, CD11c^+ ^cells, or CD19^+ ^cells) did not show any significant change of the expression of TNFα (data not shown). Although the serum TNFα concentration of BM-hIL-32 mice was comparable with that reported for human TNFα transgenic mice [1], no evident inflammation was observed in histological examination of the spleen, the joint, the intestine, the kidney, and the liver (data not shown).

We next examined the response of splenocytes of BM-hIL-32 mice to LPS stimulations. When cultured with LPS for 2 days, splenocytes of BM-hIL-32 mice showed markedly increased expression and secretion of TNFα and IL-1β (Figure 3c). Among F4/80^+ ^macrophages, CD11c^+ ^dendritic cells, CD19^+ ^B cells, CD4^+ ^T cells, and CD8^+ ^T cells from the spleen, both F4/80^+ ^macrophages and CD11c^+ ^dendritic cells showed an increased expression of TNFα and IL-1β after LPS stimulation in the splenocytes of BM-hIL-32 mice (Figure 3f and data not shown). We also observed that LPS-stimulated splenocytes of BM-hIL-32 mice showed an increased secretion of IL-6 protein (Figure 3c), and F4/80^+ ^macrophages showed an increased expression of IL-6 (Figure 3f). Notably, purified splenic CD4^+ ^T cells from BM-hIL-32 mice did not show any change in cytokine expression, including TNFα, IFN-γ, IL-1β, IL-4, IL-6, and IL-17A (data not shown). In addition, splenocyte proliferation induced by LPS or anti-CD3 antibody was no different between BM-hIL-32 mice and BM-Mock mice (data not shown).

These results suggested that the function of *in vivo *expressed IL-32β was focused on the induction of TNFα production, especially in the macrophages. Our results also suggested that *in vivo *expressed IL-32β collaborated with TLR4 signaling to induce IL-1β and IL-6 production in macrophages and dendritic cells.

### Exacerbation of TNFα-related inflammation in BM-hIL-32 mice

**Figure 4:**
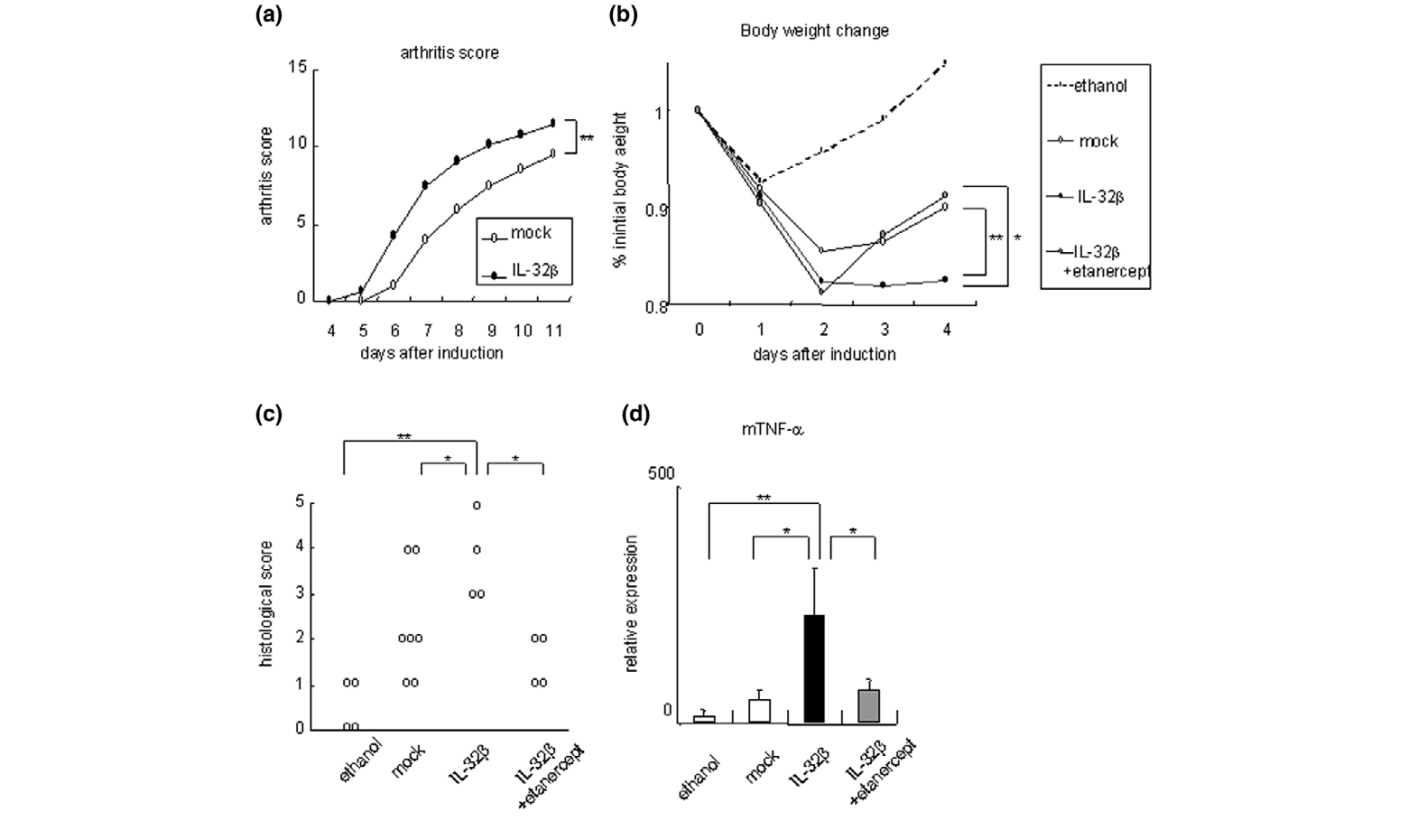
Exacerbation of murine models of tumor necrosis factor alpha-related inflammatory diseases in BM-hIL-32 mice. **(a) **Collagen-antibody-induced arthritis was induced in bone-marrow chimeric human IL-32β mice (BM-hIL-32) (*n *= 6) and bone-marrow chimeric mice of the mock group (BM-Mock) (*n *= 4). Mean arthritis scores are shown. **(b) **Body weight change after induction of trinitrobenzen sulfonic acid (TNBS)-induced colitis in BM-Mock mice (*n *= 7), in BM-hIL-32 mice (*n *= 4), and in BM-hIL-32 mice + 200 μg/day intraperitoneal administration of etanercept (*n *= 4). Control mice (*n *= 5) were administered only 50% ethanol with PBS. Percentage of initial body weight is shown. **(c) **Histological scores of TNBS-induced colitis. **(d) **Relative expression of mouse tumor necrosis factor alpha (mTNFα) in the colon of TNBS-induced colitis mice. *P < 0.05, **P < 0.01, BM-hIL-32 mice versus BM-Mock mice or BM-hIL-32 mice + etanercept.

We next examined the association of *in vivo *expressed IL-32β with TNFα-related inflammation. We prepared two kinds of murine models of inflammatory diseases – collagen antibody-induced arthritis and TNBS-induced colitis. We induced arthritis by administration of monoclonal antibodies to type II collagen and administration of LPS to BM-hIL-32 mice. After administration of LPS, more severe arthritis developed in BM-hIL-32 mice than in BM-Mock mice in the early phase of the disease (Figure 4a). This result was consistent with the *in vitro *data, which showed that LPS stimulation induced a larger amount of TNFα from splenocytes of BM-hIL-32 mice.

TNBS-induced colitis is a model of IBDs, in which TNFα plays an important role. BM-hIL-32 mice showed more severe loss of body weight than BM-Mock mice after the administration of TNBS (Figure 4b). The histological scores were significantly higher in BM-hIL-32 mice than in BM-Mock mice (Figure 4c). The expression of hIL-32β mRNA was clearly increased in the inflamed intestinal lesions of BM-hIL-32 mice but could not be detected in BM-Mock or control mice by quantitative PCR (data not shown).

Human TNF receptor p80 Fc fusion protein, known as etanercept, neutralized the action of mouse TNFα and ameliorated disease progression in collagen-immunized mice [8,33]. Although etanercept is reported as less effective in treating Crohn's disease, the efficacy of etanercept in treating refractory Crohn's disease patients has been demonstrated [34,35]. We confirmed the efficacy of an increased dose of etanercept to TNBS-induced colitis C57BL/6 mice as a preliminary study (data not shown). When etanercept was administered to TNBS-treated BM-hIL-32 mice just after the onset of colitis, the severity of body weight loss was ameliorated (Figure 4b). *In vivo *expressed IL-32 was therefore supposed to play an important role in the exacerbation of colitis, in part through the TNFα-inducing effect. The expression of TNFα was markedly increased in the rectal tissues of BM-hIL-32 mice, compared with BM-Mock mice or with etanercept-treated mice (Figure 4d). In this way, TNFα-related inflammation was exacerbated by overexpression of hIL-32β in the mouse model, and the proinflammatory effects of hIL-32β were demonstrated in the *in vivo *model.

### Exacerbation of collagen-induced arthritis by transfer of IL-32β-transduced CD4^+ ^T cells

**Figure 5:**
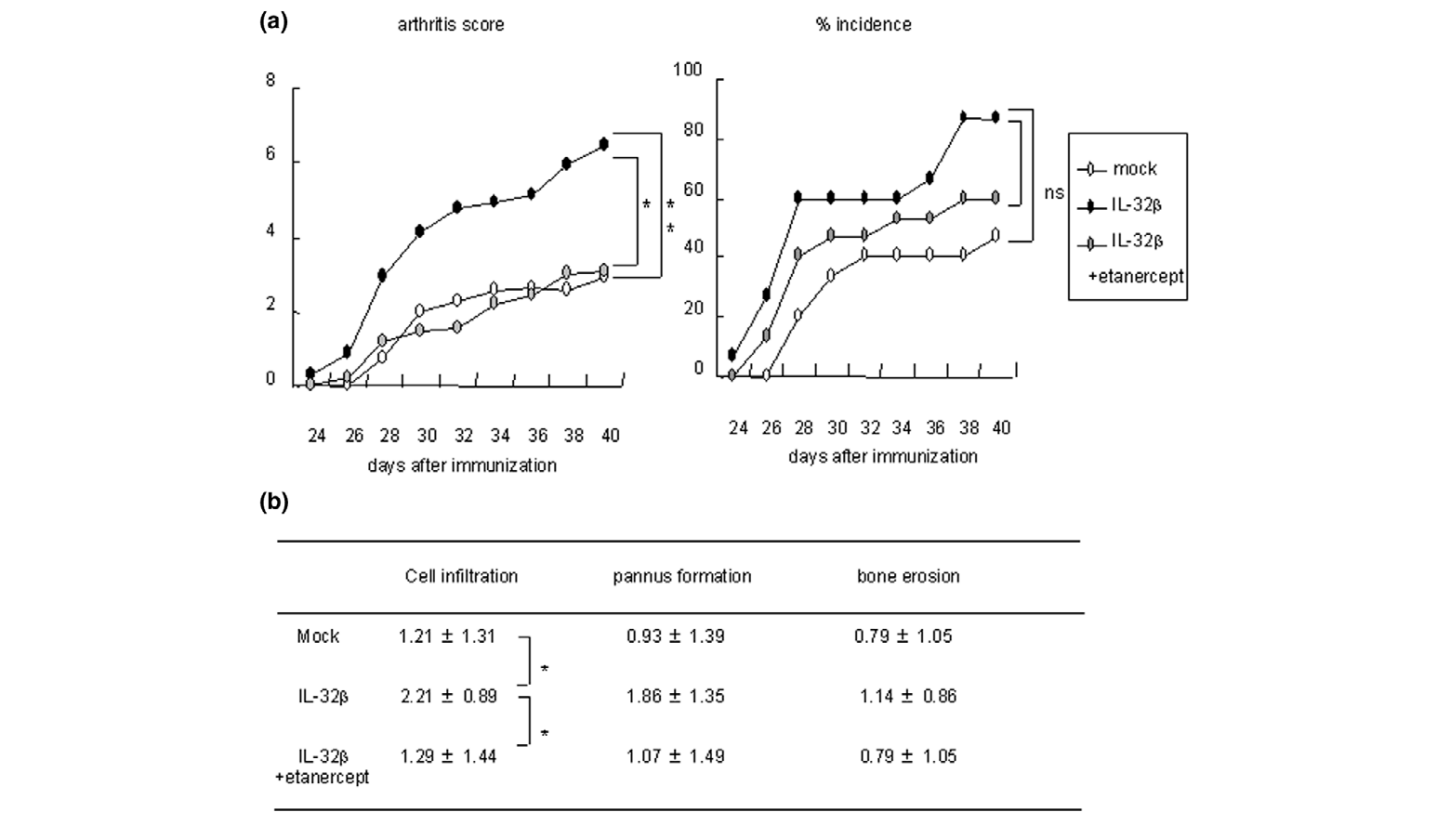
Transfer of human IL-32β-transduced CD4^+ ^T cells exacerbated collagen-induced arthritis. Human IL-32β-transduced CD4^+ ^T cells were transferred to collagen-immunized mice before the onset of arthritis (day 23). In one group (IL-32β + etanercept group), 50 μg/day etanercept was administered intraperitoneally for 14 days after transfer of CD4^+ ^T cells. Each group consisted of 14 mice. **(a) **Arthritis scores and the percentage incidence of arthritis. **(b) **Cell infiltration, pannus formation, and bone erosion in CIA mice are quantified. Histological scores are shown as the mean ± standard deviation. *P < 0.05, **P < 0.01, IL-32β group versus mock group or IL-32β + etanercept group. ns, not significant.

Since synovial-infiltrated lymphocytes strongly expressed IL-32, and peripheral CD4^+ ^T cells significantly expressed IL-32, we supposed CD4^+ ^T cells to be one of the important sources of IL-32 in the pathogenesis of inflammatory arthritis. To examine the proinflammatory effects of IL-32 produced by CD4^+ ^T cells, we transduced the hIL-32β gene to CD4^+ ^T cells with a retrovirus vector. We transferred these cells to bovine type II collagen-immunized mice before the onset of arthritis. The mice group to which the hIL-32β-transduced CD4^+ ^T cells had been transferred developed arthritis earlier than the mock group of mice and showed significantly higher arthritis scores (Figure 5a). Histological investigation of the joints showed significantly severe cell infiltration in the hIL-32β group of mice (Figure 5b). In this way, hIL-32β produced by CD4^+ ^T cells exacerbated arthritis in the mouse model.

In addition, a TNFα blockade by etanercept canceled the proarthritic effects of hIL-32β according to the clinical and pathological scores (Figure 5). IL-32-producing CD4^+ ^T cells were therefore supposed to play an important role in the exacerbation of the inflammatory arthritis, in part through a TNFα-inducing effect. The proinflammatory effects of IL-32 were therefore generally dependent on the TNFα-inducing effect in these mouse models of inflammatory diseases.

## Discussion

TNFα is a potent proinflammatory cytokine related to the pathogenesis of inflammatory diseases such as RA and IBDs [5,6,11]. The precise mechanism of TNFα induction in the inflammatory diseases, however, is still unclear. We have shown in the present article that *in vivo *expression of the novel cytokine hIL-32 induced TNFα production, and that overexpressed IL-32β significantly exacerbated the mouse model of arthritis and colitis. These results suggest that IL-32 plays an important role in the exacerbation of inflammatory diseases.

IL-32 has been reported an inducer of TNFα and other inflammatory cytokines *in vitro *[13]. Joosten and colleagues reported that the magnitude of IL-32 expression in the synovial tissues was related to the RA severity, and that recombinant hIL-32γ induced the joint inflammation in wild-type mice, which was suppressed in TNFα-deficient mice [16]. The *in vivo *effects and targets of IL-32, however, are still under examination. Moreover, the question of whether IL-32 plays a pathological role in animal models other than arthritis has not been addressed. Although the IL-32 receptor or mouse analog of IL-32 have not so far been reported, hIL-32 had biological activities on a mouse cell line and evoked joint inflammation in mice [13,16]. We therefore examined the *in vivo *effects of hIL-32β on bone marrow chimeric mice. We demonstrated the strong association *of in vivo *expressed IL-32 with TNFα production in the splenocytes, especially F4/80^+ ^macrophages. Splenocyte proliferation to the anti-CD3 antibody or LPS stimulation was not affected by the *in vivo *expression of IL-32β (data not shown). The CD4^+ ^T cells did not change cytokine expression in the presence of IL-32β. Therefore IL-32β had effects on macrophages rather on than T cells *in vivo*, and the *in vivo *roles of IL-32β were mainly to induce other inflammatory cytokines rather than to activate the proliferation of the immune cells. In the present study, we also demonstrated that the *in vivo *overexpression of hIL-32β resulted in the exacerbation of other mouse models of TNFα-related diseases – collagen-induced arthritis and hapten-induced colitis. In addition, these exacerbating effects of IL-32 were blocked by TNFα blockage, which was consistent with Joosten and colleagues' work [16].

IL-1 and IL-6 are also crucial cytokines in arthritis [4]. Injection of IL-1 into the normal joints of rabbits has caused severe arthritis [36]. IL-1RA-deficient mice developed chronic inflammatory arthritis [37,38]. Anti-IL-1 antibody and IL-1 deficiency ameliorated the mouse model of arthritis [39-41]. We have shown that the expression and secretion of IL-1β and IL-6 was increased in LPS-stimulated splenocytes from BM-hIL-32β mice. IL-1β was expressed in CD11c^+ ^dendritic cells, and IL-6 was expressed in F4/80^+ ^macrophages after LPS stimulation. IL-32 therefore induced IL-1β and IL-6 secretion in collaboration with TLR4 stimulation in these cells. In parallel withthe *in vitro *effect of LPS, disease models of BM-hIL-32 mice were exacerbated in response to anticollagen antibodies and LPS stimulation or TNBS administration. These results strongly suggest that IL-32 functions in cooperation with other inflammatory signals *in vivo*. This induction of these proinflammatory cytokines may be one of the important mechanisms of IL-32 leading towards inflammation. Although the previous report did not demonstrate the synergizing effect of hIL-32 with Toll-like receptor signaling *in vitro *[42], our results suggest that continuous exposure to hIL-32 in relatively low concentrations would have an influence on Toll-like receptor signaling of splenocytes *in vivo*. Further studies are needed to clarify the relationship between IL-32 and Toll-like receptor signaling, however, and further studies are necessary for discerning the actual mechanisms of IL-32 in the development of inflammatory diseases.

Moreover, we demonstrated a reciprocal relationship between TNFα and IL-32. TNFα induced the reciprocal expression of IL-32 in various kinds of cells (namely CD4^+ ^T cells, MoDCs, and synovial fibroblasts). We suppose that a positive feedback system between TNFα and IL-32 promotes the tissue inflammation in the synovium and the intestinal epithelium. In this way, IL-32 has a close relationship with the proinflammatory cytokines, especially TNFα, and this relationship may be one of the main mechanisms by which IL-32 promotes inflammation. Indeed, the proinflammatory effects of hIL-32β on collagen-induced arthritis mice and TNBS-induced colitis mice were, in part, canceled by a TNFα blockade.

It was reported that human TNFα transgenic mice spontaneously developed inflammatory arthritis [1]. Although the serum TNFα concentration of BM-hIL-32 mice was comparable with those of reported human TNFα transgenic mice [43], no inflammatory change was observed in histological examination of the joints (data not shown). It is suspected that this different outcome occurred because BM-hIL-32 mice expressed the transduced cytokine only in bone-marrow-derived cells, not in fibroblasts or chondrocytes of the joints. There would therefore be no initiation of inflammation in the joints without the infiltration of bone-marrow-derived cells expressing hIL-32.

The source of IL-32 remains unsolved. In our experiment, T cells played a principal role in IL-32 production. In contrast to B cells and monocytes, T cells expressed IL-32 mRNA in a resting state. Moreover, activated T cells had the capacity to induce IL-32 mRNA expression in B cells and monocytes. In addition to TCR stimulation, IL-32 mRNA expression in CD4^+ ^T cells is induced by various stimuli of inflammatory cytokines related to RA. IL-12 + IL18 stimuli, IL-23 stimuli, and TNFα stimuli increased IL-32 mRNA expression in CD4^+ ^T cells. Since the mRNA expression of IL-32 was induced by either type of stimulation, IL-32 may be associated with the pathological roles of various dendritic cell-derived cytokines (namely IL-12, IL-18, and IL-23) in inflammatory diseases. These results suggested the capacity of CD4^+ ^T cells to produce IL-32 in response to a wide range of stimuli.

T cells are reported as important mediators in inflammatory diseases, such as RA and IBDs. In terms of genetics, MHC class II genes, especially the HLA-DR1 and DR4 subtypes, are associated with RA sensitivity [44], and HLA-DRB1 is associated with Crohn's disease sensitivity [45]. In the mouse model of colitis, Th1 cells were reported as important in the pathogenesis of colitis. The colons of TNBS-treated mice were marked by infiltration of CD4^+ ^T cells exhibiting a Th1 pattern of cytokine secretion. Administration of anti-IL-12 antibodies led to a striking improvement of TNBS-induced colitis [46].

In animal models of arthritis, the transfer of CD4^+ ^T cells from SKG mice and IL-1Ra-deficient mice has evoked arthritis in the recipient mice [38,47]. In particular, IL-17 production from activated T cells is required for the development of destructive arthritis in IL-1Ra-deficient mice [48]. Like the CD4^+ ^T cells from these animal models, IL-32β-producing CD4^+ ^T cells exacerbated collagen-induced arthritis. In RA synovium, IL-32 is principally expressed in infiltrated lymphocytes, which usually contain activated T cells [49]. We therefore speculate that CD4^+ ^T cells play an important role in the exacerbation of inflammatory arthritis by means of IL-32 secretion. Notably, IL-32 mRNA expression was detected in the synovial-infiltrated lymphocytes. We supposed that IL-32-producing lymphocytes infiltrating the inflamed synovium participate in the production of TNFα in the RA synovium.

This result does not exclude the possibility that a relatively low amount of IL-32 is expressed in the synovial membranes of RA patients, because the sensitivity of *in situ *hybridization is limited [50]. In the previous study, the synovial lining cells of RA patients were stained by the anti-IL-32 antibody [16]. We assumed that the phase or type of synovial inflammation was different between Joosten and colleagues' patients and our patients. In Joosten and colleagues' report, the synovial samples were obtained by percutaneous needle biopsy, and it is suspected that their RA patients had active disease [16]. Our samples, however, were obtained from patients in the progressed stage during total joint replacement surgery. In the severely damaged joint, the cytokine-producing functions of synovial lining cells are impaired [51]. We therefore suspected that the expression of IL-32 could not be detected in the synovial lining cells by *in situ *hybridization in our study. Another explanation is the difference of types of RA. The distribution of IL-32 expression in synovial tissues may be dependent on the type of RA, a matter that needs to be examined further.

## Conclusion

IL-32 mRNA was expressed mainly in the lymphoid tissues and in a broad range of immune cells, including CD4^+ ^T cells. In RA patients, abundant IL-32 mRNA expression was observed in the synovial-infiltrated lymphocytes. Splenic macrophages of hIL-32β-overexpressed mice showed increased expression and secretion of TNFα and IL-6, and splenic dendritic cells showed increased expression and secretion of IL-1β in response to LPS stimulation. Overexpression of hIL-32β also resulted in the exacerbation of collagen antibody-induced arthritis and TNBS-induced colitis. IL-32β-producing CD4^+ ^T cells significantly exacerbated inflammatory arthritis in the mouse model. The effects of IL-32 in different disease models were almost canceled by TNFα blockade.

This is the first study that demonstrated the *in vivo *cytokine-inducing effects of IL-32. In addition, the reciprocal induction between IL-32 and TNFα was also demonstrated in many types of cells. IL-32 therefore has a close relationship with TNFα, contributes to the exacerbation of inflammatory diseases, and could be a new therapeutic target of these inflammatory diseases.

## Abbreviations

BM-hIL-32 = overexpression model of human IL-32β model by bone marrow transplantation; Con A = concanavalin A; ELISA = enzyme-linked immunosorbent assay; FCS = fetal calf serum; GFP = green fluorescent protein; H & E = hematoxylin and eosin; hIL-32 = human interleukin-32; IBD = inflammatory bowel disease; IL = interleukin; LPS = lipopolysaccharide; mAb = monoclonal antibody; MACS = magnetic-activated cell sorting; MHC = major histocompatibility comprex; MoDC = monocyte-derived dendritic cell; PBMC = peripheral blood mononuclear cell; PBS = phosphate-buffered saline; PCR = polymerase chain reaction; RA = rheumatoid arthritis; RT = reverse transcriptase; TNBS = trinitrobenzen sulfonic acid; TCR = T-cell receptor; TNFα = tumor necrosis factor alpha.

## Competing interests

The authors declare that they have no competing interests.

## Authors' contributions

HS carried out the molecular and animal experiments, performed the statistical analysis, and drafted the manuscript. KF supervised the study design, the statistical analysis, and the writing of the manuscript. YY and AO carried out the cell culture experiments. TS prepared the human samples of synovial tissues and cells. YK performed the quantitative PCR. KY supervised the study design and gave valuable advice to HS. All authors read and approved the final manuscript.

## Figures and Tables

**Table 1:** Primers used in the real-time PCR

Human IL-32	Sense	5'-TGAGGAGCAGCACCCAGAGC-3'
	Antisense	5'-CCGTAGGACTGGAAAGAGGA-3'
Human TNFα	Sense	5'-GTCTCCTACCAGACCAAG-3'
	Antisense	5'-CAAAGTAGACCTGCCCAGACTC-3'
Human β-actin	Sense	5'-TTCCTGGGCATGGAGTCCT-3'
	Antisense	5'-AGGAGGAGCAATGATCTTGATC-3'
Mouse TNFα	Sense	5'-CATCTTCTCAAAATTCGAG-3'
	Antisense	5'-TGGGAGTAGACAAGGTACAACCC-3'
Mouse IL-1β	Sense	5'-CAACCAACAAGTGATATTCTCCATG-3'
	Antisense	5'-GATCCACACTCTCCAGCTGCA-3'
Mouse IL-6	Sense	5'-CACTTCACAAGTCGGAGGCTTA-3'
	Antisense	5'-GCAAGTGCATCATCGTTGTTG-3'
Mouse β-actin	Sense	5'-AGAGGGAAATCGTGCGTGAC-3'
	Antisense	5'-CAATAGTGATGACCTGGCCGT-3'

TNFα, tumor necrosis factor alpha.
